# Editorial: Age and health management practices: impact on modern organizations

**DOI:** 10.3389/fpsyg.2024.1401398

**Published:** 2024-04-15

**Authors:** Vlado Dimovski, Katarina Vukojević, Renata Pecotić, Simon Colnar

**Affiliations:** ^1^School of Economics and Business, University of Ljubljana, Ljubljana, Slovenia; ^2^School of Medicine, University of Split, Split, Croatia

**Keywords:** age management, health management, aging, modern organization, aging workforce, mental wellbeing, chronic disease, health care

The Research Topic “*Age and health management: impact on modern organizations*,” explores how we can consider aging as a global phenomenon and how it is the reality nowadays for organizations and society as it is happening at an unprecedented pace. The collection of original research articles contains a vast range of topics related to aging such, as stress and suicidal ideation within the baby boomer generation, gaining insight into health care in the future community and examination of the ecosystem of human capital in care homes. Along with innovative approaches toward online physical exercise of older adults, this Research Topic focused on depression and influencing factors in middle-aged and elderly patients who are dealing with chronic diseases.

We can argue that aging is a fascinating topic, as it deals with different challenges relevant at the individual, organizational and overall societal levels (Salminen et al., 2019). The worldwide aging of the population is changing much of the existing economic and social order, with significant consequences for different fields such as production, consumption, health care, and the labor market (Castro-Conde and Gutiérrez de Rubalcava, 2018; Chen et al., 2018). A growing body of literature enables a solid basis for understanding the reality of aging population and its accompanying aging of the workforce. However, there has been little direct research into how modern organizations could and should utilize the benefits of age diversity in contemporary organizations (Sammarra et al., 2017). Despite the growing relevance of age and health management, research from different organizational and societal perspectives and its implications for modern organizations and society remains scarce. Therefore, we argue that more research is necessary to investigate how organizations can adapt and implement their age and health management practices, where age management practices can focus on recruitment policies, retirement plans, and training. Health management practices could pay attention to overall health and mental wellbeing, including an emphasis on chronic diseases and different mental health aspects in the workplace and societal context. This approach will enable modern organizations to ensure that their older employees can still utilize their strengths to perform successfully in the organization while remaining healthy (Dimovski et al., 2022), which will also have an overall positive impact on the societal level.

The Research Topic “*Age and health management: impact on modern organizations*,” has five published articles and all within the framework of original research articles.

The first article by Jeong, entitled “*Stress and suicidal ideation in Korean baby boomers: the mediating effect of mindfulness and meaning in life*,” is an original research article that sheds additional light on the relationship between stress and suicidal ideation. Its novelty includes the effect of mindfulness and the meaning of life on the relationship between stress and suicidal ideation. The original research was conducted within the context of Korean baby boomers. According to the authors, the findings indicate that if interventions that are directed at baby boomers can successfully improve their mindfulness and extend their meaning in life, suicidal ideation will decrease within their population.

The second article by Zhou et al., entitled “*Health care in future community: innovatively discover and respond to the needs of today's seniors*,” is an original research that focuses on the emergence and application of emerging technologies that have accelerated the integration of traditional social structures with new technologies, leading to the inception of the “Future Community” as an innovative urban unit. The findings show that the means of community health care for older adults are gradually being upgraded, and the demands are shifting. This study offers three contributions. Firstly, technological innovation and intelligent approaches have the potential to influence the quality of health care in these communities positively. Secondly, allocating health care resources within communities can have a beneficial effect on the psychological wellbeing of seniors. Thirdly, actively involving seniors in community life and governance can elevate their self-worth.

The third article by Kejžar and Turunen, entitled “*The ecosystem of human capital in care homes*,” is a qualitative original research study that addresses the essential yet often overlooked knowledge transfer experiences within care homes. The study aims to explore barriers and facilitators for knowledge transfer that are crucial for creating new knowledge services and enhancing care quality for older individuals. The authors examined how knowledge transfer within care homes was facilitated through continuous training, diverse communication channels, and mentoring. Collaboration with relatives improved understanding of resident preferences and habits and overall enhanced the quality of care. The research reveals how this collaborative effort allowed mutual learning and knowledge transfer from the care homes to the broader community.

The fourth article by Lin et al., entitled “*Analysis of depression status and influencing factors in middle-aged and elderly patients with chronic diseases*,” is an original research article that offers new knowledge on the prevalence of depression and its influencing factors in middle-aged and elderly patients with chronic disease. The authors noted that depressive symptoms are common in Chinese older adults with chronic diseases. They need regular assessment and intervention, especially those with multiple diseases, female, rural, alone, impaired, poor sleep, or poor economy. These high-risk elders also need family, medical, and social support and care.

The fifth article by Peterlin et al., entitled “*Older adults' perceptions of online physical exercise management*,” is an original research article that discusses the challenges of digitally transforming physical exercises for older adults to be performed in virtual environments as a long-term proactive strategic initiative in response to the global aging society and technological development trend. Utilizing a focus group conducted by the Jožef Stefan Institute, the authors were able to identify the skills and wellbeing gained and identify factors that influence success with online exercises for older adults on the individual and organizational levels. The study results are intended to benefit health management practices and theory in the work environment to ensure that older workers can still utilize their strengths to perform successfully while remaining healthy. Online physical exercises tailored to older adults' needs and specifications could be provided as part of corporate wellness programs in organizations.

In conclusion, aging will influence every national context, modern organization, and economy. Thus, addressing these critical challenges by applying appropriate age and health management practices in contemporary organizations becomes essential and will benefit society.

## Author contributions

VD: Writing – original draft, Writing – review & editing. KV: Writing – original draft, Writing – review & editing. RP: Writing – original draft, Writing – review & editing. SC: Writing – original draft, Writing – review & editing.
